# Clues to Occult Cancer in Patients with Ischemic Stroke

**DOI:** 10.1371/journal.pone.0044959

**Published:** 2012-09-12

**Authors:** Suk Jae Kim, Jae Hyun Park, Mi-Ji Lee, Yun Gyoung Park, Myung-Ju Ahn, Oh Young Bang

**Affiliations:** 1 Department of Neurology, Samsung Medical Center, Sungkyunkwan University School of Medicine, Seoul, Korea; 2 Department of Internal Medicine, Samsung Medical Center, Sungkyunkwan University School of Medicine, Seoul, Korea; 3 Department of Hemato-oncology, Samsung Medical Center, Sungkyunkwan University School of Medicine, Seoul, Korea; Innsbruck Medical University, Austria

## Abstract

**Background:**

We hypothesized that hidden malignancy could be detected in patients with cryptogenic stroke without active cancer when they showed the distinctive characteristics of cancer-related stroke.

**Methods and Findings:**

Among 2,562 consecutive patients with acute ischemic stroke, patients with cryptogenic stroke were analyzed and categorized into two groups according to the presence of active cancer: cryptogenic stroke with active cancer (cancer-related stroke, CA-stroke) group and without active cancer (CR-stroke) group. Patients with active lung cancer without stroke were also recruited for comparison purposes (CA-control). Clinical factors, lesion patterns on diffusion-weighted MRI (DWI), and laboratory findings were analyzed among groups. A total of 348 patients with cryptogenic stroke were enrolled in this study. Among them, 71 (20.4%) patients had active cancer at the time of stroke. The D-dimer levels were significantly higher in patients with CA-stroke than those with CR-stroke or CA-control (both *p*<0.001). Regarding lesion patterns, patients with CA-stroke mostly had multiple lesions in multiple vascular territories, while more than 80% of patients with CR-stroke had single/multiple lesions in a single vascular territory (*P*<0.001). D-dimer levels (OR 1.11 per 1 µg/mL increase; 95% CI 1.06–1.15; *P*<0.001) and DWI lesion patterns (OR 7.13; 95% CI 3.42–14.87; *P*<0.001) were independently associated with CA-stroke. Workup for hidden malignancy was performed during hospitalization in 10 patients who showed elevated D-dimer levels and multiple infarcts involving multiple vascular territories but had no known cancer, and it revealed hidden malignancies in all the patients.

**Conclusion:**

Patients with CA-stroke have distinctive D-dimer levels and lesion patterns. These characteristics can serve as clues to occult cancer in patients with cryptogenic stroke.

## Introduction

Cancer and cerebrovascular disease are the leading causes of mortality and morbidity worldwide among the elderly. With the development of cancer treatment over recent decades, the number of cancer patients with stroke is expected to rise. The occurrence of cerebrovascular disease is not uncommon in cancer patients; 15% of them had a thromboembolic complication during their clinical course [1]. Mechanisms underlying stroke in patients with cancer can be divided into three types: cancer-unrelated mechanisms (conventional stroke mechanisms; atherosclerosis, cardioembolism, lacunar infarction, etc.), cancer-related mechanisms, and treatment-related mechanisms [2]. It has been recently reported that embolism caused by hypercoagulopathy is the main mechanism of cancer-related stroke, especially in cancer patients without conventional stroke mechanisms [3], [4].

The association between cancer and venous thromboembolism is well established. Previous studies have demonstrated that a specific form of venous thrombosis, thrombophlebitis migrans, may appear months or even years before the signs and symptoms of cancer emerge [5]. This rare disorder is considered as a clue to the presence of hidden malignancy. In addition, it has been well known that deep venous thrombosis, which is a more common form of venous thrombosis, or pulmonary embolism also lead clinicians to suspect occult cancer [6]–[8]. However, it is not known if the occurrence of ischemic stroke, which is suspected to be related to cancer, should lead clinicians to suspect occult malignancy.

We investigated the clinical, radiological, and laboratory characteristics of cancer-related stroke among patients without conventional stroke mechanisms. We hypothesized that hidden malignancy could be detected in cryptogenic stroke patients without overt cancer when they showed the distinctive characteristics of cancer-related stroke.

## Methods

### Patients and Grouping

This retrospective analysis was conducted using data collected from a prospective registry of patients who presented at our institute between December 2006 and October 2011. The inclusion criteria for this study were: (a) subjects with focal neurologic deficits that presented within 7 days of the onset of symptoms, (b) subjects with acute ischemic lesions on diffusion-weighted MRI (DWI), and (c) subjects undergoing diagnostic workups, including vascular and cardiologic studies. Among 2562 patients who met the inclusion criteria, the following patients were excluded from the study: (1) those who had one or more evident stroke etiologies (conventional stroke mechanisms) by the Stop Stroke Study Trial of Org 10172 in Acute Stroke Treatment (SSS-TOAST) classification [9], (2) those who had not undergone MRI or for whom no relevant lesions were seen on DWI, (3) those who had incomplete workups for stroke etiology (either vascular or cardiologic studies), (4) those in whom plasma D-dimer was not examined within 24 hours from admission, (5) those who had a history of recent surgery, myocardial infarction, or deep vein thrombosis, or any signs of infectious or immunological diseases, which may influence plasma D-dimer levels, or (6) those who had primary intracranial tumor.

**Figure 1:**
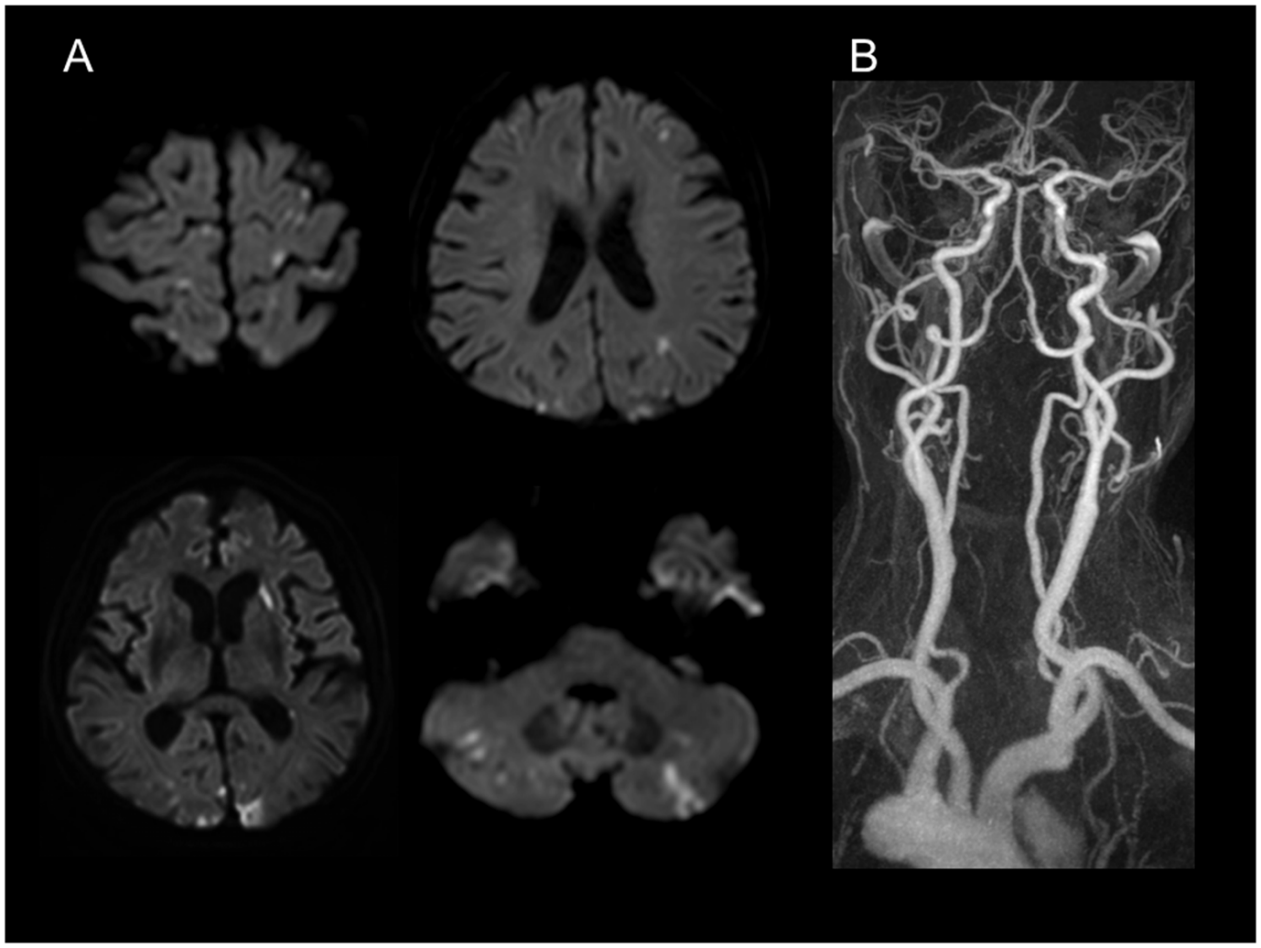
A typical example of cryptogenic stroke with active cancer (cancer-related stroke). The patient was a 55-year-old female, who was diagnosed with metastatic adenocarcinoma of lung one year ago. Neurologic examination revealed motor aphasia, right-central facial paralysis, and right hemiparesis. Initial D-dimer levels were 20.56 µg/mL. Diffusion-weighted MRI (A) shows multiple lesions involving multiple vascular territories. MR angiography (B) reveals no significant stenosis or occlusion of the craniocervical arterial vasculature. Comprehensive workup including transesophageal echocardiogram and 24-hour Holter monitoring demonstrated no sources of cardioembolism.

Since we excluded all the patients with conventional stroke mechanisms, subjects included in this study were considered to have cryptogenic stroke. Patients were classified into two groups according to the presence of active cancer at the time of stroke: (1) the cryptogenic stroke with active cancer (cancer-related stroke, CA-stroke group) and (2) the cryptogenic stroke without active cancer (CR-stroke group). Active cancer was defined as a diagnosis of cancer within 6 months prior to enrollment, any treatment for cancer within the previous 6 months, or recurrent or metastatic cancer, as previously described [10]. A typical example of CA-stroke is presented in Figure 1. As a disease control group, patients with locally advanced lung cancer or distant organ metastases, and without a history of stroke were recruited for comparison purposes (CA-control group). All patients gave written informed consent, and the institutional review board in Samsung Medical Center approved this study.

### Workups

Demographic and clinical data were collected at admission and included sex, age, and vascular risk factors including hypertension, diabetes mellitus, hyperlipidemia, coronary artery disease, atrial fibrillation, and smoking habits. Routine laboratory data were collected for all patients and included fibrinogen and D-dimer within 24 hours after admission. The plasma levels of D-dimer were determined by immunoturbidimetry on a STA-R automated analyzer (Diagnostica Stago, Asnieres, France) (reference values in our laboratory ≤0.5 µg/mL).

Hemostatic markers of prothrombotic tendency, including antiphospholipid antibodies (anticardiolipid antibody, lupus anticoagulants, and β2-glycoprotein-1 antibody) were measured in patients younger than 50 years to exclude hypercoagulable state caused by other than cancer. Diagnostic work-up to detect other rare caused of stroke (e.g. vasculitis) was performed in selected patients based on the attending physician’s discretion. All patients underwent transthoracic echocardiography, 12-lead electrocardiography, Holter and/or telemetry monitoring, and brain MRI. In addition, transcranial Doppler or transthoracic echocardiographic bubble test was underwent in selected patients in whom DWI pattern suggested embolic stroke mechanism.

The typical MRI protocol included at least DWI, fluid-attenuated inversion recovery (FLAIR), and vascular images (3D time-of-flight MR angiography (MRA) and contrast-enhanced MRA including extracranial internal cerebral artery and vertebral artery). The DWI parameters were as follows: repetition time (TR), 2550 ms; echo time (TE), 75 ms; matrix, 128×128; 3 directions; field of view (FOV), 24 cm; section thickness, 5 mm; and intersection gap, 2 mm. DWI was obtained with b values of 0 and 1000 s/mm^2^. We analyzed DWI data in all patients. DWI patterns were classified as single/multiple lesions involving a single vascular territory and multiple leisons involving multiple vascular territories. The involvement of multiple vascular territories was defined by multiple ischemic lesions in the: 1) unilateral anterior and posterior circulation, 2) bilateral anterior circulation, or 3) bilateral anterior and posterior circulation.

### Statistical Analyses

Statistical analyses were performed using a commercially available software package (PASW version 18.0; SPSS Inc, Chicago, Ill, USA). All data are presented as the median (25^th^–75^th^ percentile) or number (percentage). The Shapiro-Wilk test was used to test for normal distribution of continuous variables. Since the distributions were not normal (*P*<0.05), Mann-Whitney U test was conducted to compare continuous variables between groups. Pearson’s chi-square or Fisher’s exact test was used to compare categorical variables. We used the Bonferroni correction to account for multiple tests.

The optimal D-dimer value for diagnosis of CA-stroke among patients with cryptogenic stroke was established with a receiver operating characteristic (ROC) scatter plot (MedCalc For Windows, version 9.3, MedCalc software, Mariakerke, Belgium). An alternative way to establish an optimal cutoff value for a test is to determine the optimal decision point from an ROC curve, whereby equal weight is given to the sensitivity and specificity of the test. The sum of the sensitivity and specificity values is highest at this point. To calculate the sensitivity, specificity, and positive and negative predictive values of diagnostic tests, we used this cutoff point.

Multivariable logistic regression analysis was performed to predict the independent contribution of factors in CA-stroke vs. CR-stroke. Variables significant at *P*<0.2 on univariable analyses were considered explanatory variables and were entered together into multivariable models. Plasma levels of D-dimer were treated as a continuous variable (Model 1) or categorized as quartiles (Model 2) in multivariable analyses. A *p*-value <0.05 was considered statistically significant.

## Results

During the study period, a total of 348 patients with cryptogenic stroke (217 men and 131 women) were ultimately enrolled in this study. Among them, 71 (20.4%) patients had active cancer at the time of presentation of stroke. The mean patient age was 61 years old (standard deviation, 15; full range, 18–94 years). As a disease control group, 33 patients with active lung cancer without a history of stroke were also included.

Baseline characteristics of patients with CA-stroke, CR-stroke, and CA-control are presented in Table 1. The median time interval between stroke onset and the diagnosis of cancer was 5 months (25^th^–75^th^ percentile, 2–25 months) among patients with CA-stroke. In terms of the primary cancer, lung cancer was most frequently observed, followed by hepatobiliary, gastrointestinal, and breast-gynecologic malignancies. The majority of patients had adenocarcinoma as the histologic subtype and systemic metastasis. Approximately one third of CA-stroke patients had received chemotherapy possibly causing coagulopathy (all cisplatin treatments) within 6 months of stroke onset.

**Table 1:** Baseline characteristics.

	Cancer-related stroke(N = 71)	Cryptogenic stroke without active cancer (N = 277 )		Active lung cancer without stroke (N = 33)	
**Male sex**	41 (57.7%)		176 (63.5%)		18 (54.5%)
**Age, years**	63 (49–73)		63 (55–71)		57 (52–64)
**Vascular risk factors**					
** Hypertension**	20 (28.2%)		131 (47.3%)†		9 (27.3%)
** Diabetes**	13 (18.3%)		48 (17.3%)		5 (15.2%)
** Hyperlipidemia**	5 (7.0%)		73 (26.4%)‡		3 (9.1%)
** Current smoking**	13 (18.3%)		66 (23.8%)		7 (21.2%)
** Coronary artery disease**	1 (1.4%)		19 (6.9%)		1 (3.0%)
**Laboratory findings**					
** D-dimer, µg/mL**	10.67 (3.08–25.67)		0.45 (0.29–1.01)‡		0.67 (0.40–1.35)‡
** Fibrinogen, mg/dL**	337 (213–500)		306 (259–380)		N/A
**DWI patterns**					
** Single vascular territory**	17 (23.9%)		229 (82.7%)		N/A
** Single lesion**	5		143		N/A
** Multiple lesions**	12		86		N/A
** Multiple vascular territories**	54 (76.1%)		48 (17.3%)‡		N/A
**Pre-stroke medication**					
** Antiplatelet agents**	7 (9.9%)		54 (19.5%)		5 (15.2%)
** Anticoagulants**	4 (5.6%)		3 (1.1%)		0 (0%)
**Cancer profiles**					
** Time interval between stroke onset and the diagnosis of cancer, months**	5 (2–25)		N/A		N/A
** Primary cancer type**					
** Lung**	29 (40.8%)		N/A		33 (100%)
** Gastrointestinal**	11 (15.5%)		N/A		N/A
** Hepatobiliary**	18 (25.4%)		N/A		N/A
** Breast-gynecologic**	7 (9.9%)		N/A		N/A
** Others**	6 (8.5%)		N/A		N/A
** Systemic metastasis**	47 (66.2%)		N/A		20 (60.6%)
** Adenocarcinoma**	48 (67.6%)		N/A		26 (78.8%)
** Chemotherapy causing coagulopathy**	23 (32.4%)		N/A		12 (36.4%)
** Cisplatin**	23		N/A		12
** Methotrexate**	0		N/A		0
** L-asparaginase**	0		N/A		0
** Bevacizumab**	0		N/A		0

Test for differences vs. cancer-related stroke: * *P*<0.05, † *P*<0.01, ‡ *P*<0.001.

DWI indicates diffusion-weighted MRI; N/A, not applicable or data not available.

The CA-control group had similar characteristics in terms of demographics, vascular risk factors, antithrombotic use, and chemotherapy compared to the CA-stroke group. In addition, the proportion of patients with systemic metastasis and adenocarcinoma was also comparable between the two groups. Regarding laboratory findings, the plasma levels of D-dimer were significantly higher in patients with CA-stroke than in those with CA-control. D-dimer levels were the only factor that showed a significant difference between those two groups (*P*<0.001).

**Figure 2:**
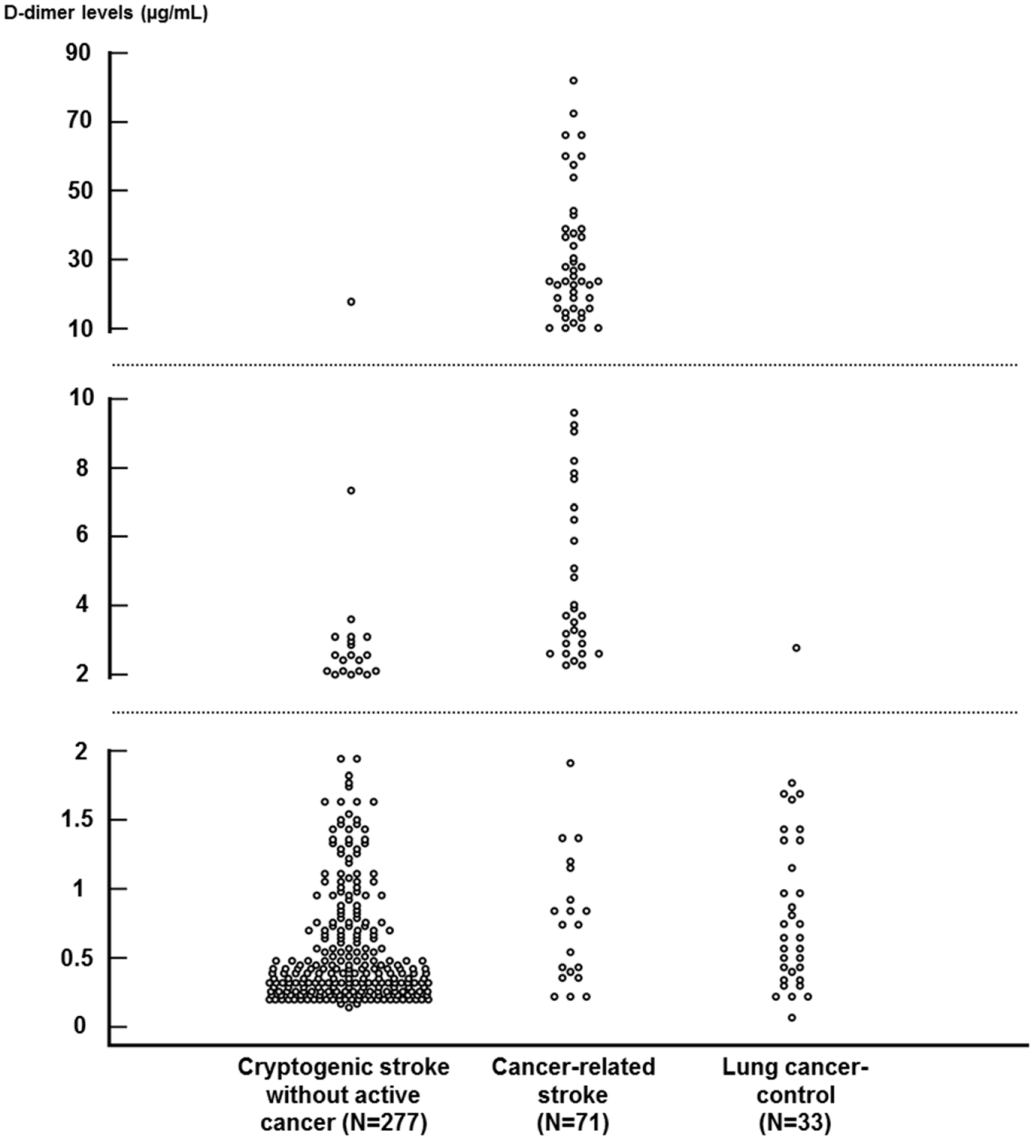
Distribution of plasma D-dimer levels in patients with cancer-related stroke (N = 71), cryptogenic stroke without active cancer (N = 277), and active lung cancer without a history of stroke (N = 33). D-dimer levels were significantly higher in the cancer-related stroke group than in the cryptogenic stroke without active cancer or lung cancer-control groups (both *P*<0.001).

The plasma D-dimer levels were also significantly higher in the CA-stroke group compared to the CR-stroke group (*P*<0.001) (Fig. 2). In terms of lesion patterns on DWI, most of the patients with CA-stroke had multiple lesions in multiple vascular territories, while more than 80% of patients with CR-stroke had single or multiple lesions in a single vascular territory (*P*<0.001). With regard to vascular risk factors, hypertension and hyperlipidemia were less prevalent in the CA-stroke group than the CR-stroke group (*P*<0.01 and *P*<0.001, respectively). Other factors including demographic profiles, a history of diabetes, smoking, and coronary artery disease, and pre-stroke medications did not differ between the two groups.

Multivariate logistic regression analysis was performed to further evaluate the independent predictors for CA-stroke vs. CR-stroke (Table 2). Plasma levels of D-dimer (per 1 µg/mL increase; OR 1.11; 95% CI 1.06–1.15; *P*<0.001 for Model 1; highest quartile versus lowest quartile; OR 4.83; 95% CI 2.04–11.46; *P*<0.001 for Model 2) and multiple lesions involving multiple vascular territories on DWI (OR 7.13; 95% CI 3.42–14.87; *P*<0.001 for Model 1; OR 4.83; 95% CI 2.04–11.46; *P*<0.001 for Model 2) were independently associated with CA-stroke. Other factors including hypertension, hyperlipidemia, smoking habit, and pre-stroke antiplatelet use did not significantly add value to CA-stroke.

**Table 2:** Multiple logistic regression analysis: Independent predictors for cancer-related stroke vs. cryptogenic stroke without active cancer.

	Crude OR	Multivariate testing			
		Model 1		Model 2	
		OR (95% CI)	*p* value	OR (95% CI)	*p* value
**Hypertension**	0.44	···	···	···	···
**Hyperlipidemia**	0.21	···	···	···	···
**Current smoking**	0.72	···	···	···	···
**Pre-stroke antiplatelet therapy**	0.45	···	···	···	···
**Lesion patterns on DWI**					
** Single vascular territory**	Ref.	Ref.	Ref.	Ref.	Ref.
** Multiple vascular territory**	15.15	7.13 (3.42–14.87)	<0.001	4.83 (2.04–11.46)	<0.001
**Plasma D-dimer levels, per 1 µg/mL increase**	1.17	1.11 (1.06–1.15)	<0.001	N/A	N/A
**Plasma D-dimer levels, µg/mL**					
** 1^st^ quartile (<0.33)**	Ref.	N/A	N/A	Ref.	Ref.
** 2^nd^ quartile (0.33–0.64)**	0.49	N/A	N/A	0.40 (0.04–4.56)	0.458
** 3^rd^ quartile (0.64–2.13)**	3.04	N/A	N/A	3.11 (0.59–16.38)	0.182
** 4^th^ quartile (≥2.13)**	104.16	N/A	N/A	58.72 (12.37–278.84)	<0.001

DWI indicated diffusion-weighted MRI; OR, odds ratio; CI, confidence interval; Ref, reference; N/A, not applicable.

**Figure 3:**
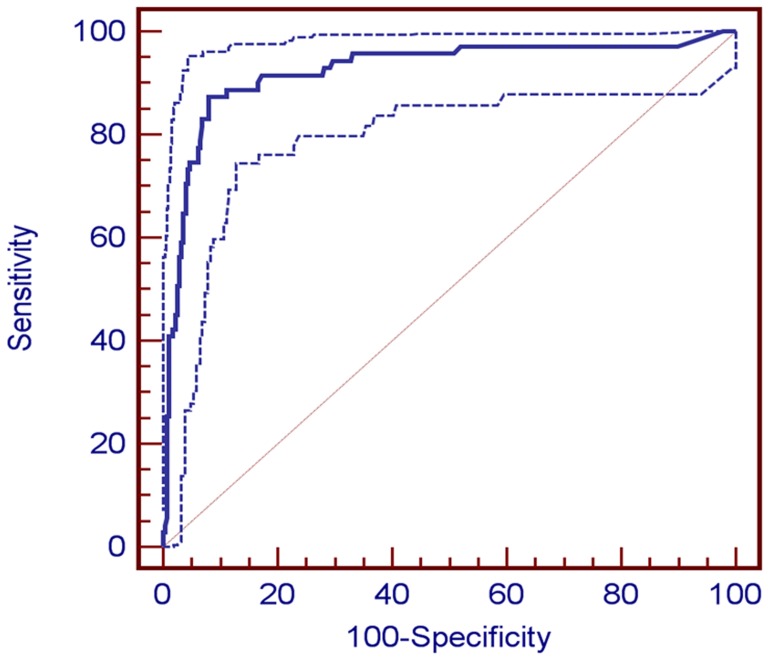
Receiver operating characteristic (ROC) curve for predicting cancer-related stroke from plasma D-dimer levels. The area under the ROC curve (AUC) ± standard error was 0.945±0.182 (*P*<0.001). The dotted lines indicate 95% confidence interval (0.916 to 0.967).

The ROC curve for diagnosing CA-stroke from plasma D-dimer levels is provided in Figure 3. The mean ± standard error of the area under curve (AUC) for D-dimer was 0.945±0.182 (*P*<0.001), indicating good overall accuracy of the test. Optimum diagnostic cut-off levels were identified from the ROC curve for D-dimer (>2.15 µg/mL). The sensitivity, specificity, and positive and negative predictive values (%) for diagnosis of CA-stroke at this level were 73.8 (95% CI 62.9–82.5), 96.6 (95% CI 93.4–98.3), 87.3 (95% CI 76.8–93.7), and 92.1 (95% CI 88.1–94.8), respectively.

Among subjects with CR-stroke at the time of stroke, there were 22 patients with D-dimer over the cut-off values. The detailed characteristics of the subjects are summarized in Table 3. Ten of those patients had multiple lesions in multiple vascular territories on DWI, which was a characteristic pattern in the CA-stroke group. Per the attending physician’s decision, they underwent workup for hidden malignancy during hospitalization, and occult cancer was ultimately found in all the patients. The primary cancer was located in lung (n = 4), gastrointestinal (n = 5), and hepatobiliary (n = 1) system. The majority had adenocarcinoma as a histologic subtype and systemic metastasis, which were similar findings to those of the CA-stroke group.

**Table 3:** Summary of detailed characteristics of subjects with D-dimer over cut-off values (2.15 µg/mL) in cryptogenic stroke without active cancer.

No.	Sex	Age	D-dimer levels(µg/mL)	DWI pattern	Occult cancer	Primary cancer type	Systemic metastasis	Adenocarcinoma
1	Female	85	2.43	MS	No			
2	Male	69	2.51	S	No			
3	Male	70	2.58	MS	No			
4	Male	50	2.63	S	No			
5	Male	68	2.87	S	No			
6	Female	76	2.97	MS	No			
7	Male	72	3.09	MS	No			
8	Female	78	3.09	S	No			
9	Female	79	3.11	MS	No			
10	Female	65	3.24	MM	Yes	Lung	No	Yes
11	Male	76	3.61	S	No			
12	Female	48	4.83	MM	Yes	Lung	No	Yes
13	Female	72	7.33	MS	No			
14	Female	90	8.21	MM	Yes	Gastrointestinal	Yes	No
15	Female	71	10.48	MM	Yes	Lung	No	No
16	Female	68	13.40	MM	Yes	Gastrointestinal	Yes	No
17	Female	69	15.88	MM	Yes	Lung	Yes	Yes
18	Male	71	16.41	MM	Yes	Gastrointestinal	Yes	Yes
19	Female	68	17.89	S	No			
20	Male	75	25.20	MM	Yes	Gastrointestinal	Yes	Yes
21	Male	61	60.00	MM	Yes	Gastrointestinal	Yes	Yes
22	Female	61	66.80	MM	Yes	Hepatobiliary	Yes	Yes

DWI indicated diffusion-weighted MRI; S, single lesion in a single vascular territory; MS, multiple lesions in a single vascular territory; MM, multiple lesions in multiple vascular territories.

Interestingly, if the ten patients who were newly found to have hidden malignancies were changed from CR-stroke to CA-stroke, the sensitivity, specificity, and positive and negative predictive values (%) are enhanced to 85.7 (95% CI 76.0–92.1), 96.6 (95% CI 93.4–98.3), 88.9 (95% CI 79.5–94.5), and 95.5 (92.1–97.5), respectively.

## Discussion

The major findings of the current study are (a) plasma D-dimer levels are significantly higher in patients with CA-stroke than in those with CR-stroke and CA-control, (b) acute ischemic lesions involving multiple vascular territories on DWI and plasma D-dimer levels are independent predictors for CA-stroke, and (c) those predictors also serve as clues to the presence of occult cancer in patients with CR-stroke.

### Distinctive Features of Cancer-related Stroke

The unveiling of underlying causes of stroke is an important issue in patients with cryptogenic stroke, because causes influence the choice of management, stroke prognosis, and risk of recurrence [11]. Certain clinical, laboratory, and radiological features could help physicians determine stroke etiology early. Among them, the pattern of ischemic lesions demonstrated by DWI has been reported to be correlated with the underlying pathogenic mechanisms of stroke or even serve as a surrogate marker of specific causes of stroke [12].

In this study, the pattern of multiple ischemic lesions extending to multiple vascular systems was an independent predictor of CA-stroke. It has been well known that multiple acute stroke lesions on DWI are presumably caused by multiple emboli [13], [14]. Our previous study using microembolic signals detected by transcranial Doppler also suggested the embolic nature of CA-stroke [3]. However, it cannot be said that the feature is unique for CA-stroke, because the same topography on DWI could also result from cardiogenic, paradoxical, and aortogenic embolic strokes [15].

The notable characteristic of CA-stroke is high plasma concentrations of D-dimer. The D-dimer levels are a direct measure of activated coagulation and have been used as a means of hypercoagulability [16]. The elevated D-dimer levels in CA-stroke suggest that hypercoagulopathy plays a major role in the pathogenesis of CA-stroke, which is a consistent finding with previous studies [3], [4], [17]. Interestingly, the D-dimer concentrations were also significantly higher in CA-stroke than CA-control, which might mean that certain trigger factors are needed in the development of CA-stroke. Recent studies demonstrated that tissue factor-bearing cancer-derived microparticles have a significant role in the pathogenesis of thrombosis in patients with cancer [18], [19]. We are investigating the possible role of the microparticles in the development of CA-stroke. Concerning the high predictability of D-dimer to diagnose CA-stroke, we think that the D-dimer levels in conjunction with lesion patterns on DWI can be used as a discriminating marker for CA-stroke in patients with cryptogenic infarction.

### Clues to Occult Cancer in Stroke Patients

Since a hypercoagulable state is a common condition in patients with cancer, the cancer patient is predisposed to the development of venous thrombosis. In this regard, the possible role of venous thrombosis as a clue to occult malignancy has been widely investigated. Studies show that superficial venous thrombosis, idiopathic deep vein thrombosis, and bilateral deep vein thrombosis correlate with subsequent cancer diagnosis [20], [21]. However, there is a paucity of data regarding the clues to hidden malignancy in patients with ischemic stroke.

In this study, we found cancers in all the patients who had compatible features with CA-stroke among the CR-stroke group. In other words, clinicians should suspect occult cancer when they diagnose ischemic stroke involving multiple vascular territories with high D-dimer levels and without identifiable causes of stroke or elevated D-dimer which are consistent with findings from one previous study [22]. Regarding location, histology, and extent of cancer, malignancies in patients with CA-stroke and hidden malignancies in patients with CR-stroke had similar features. Adenocarcinoma in the lung, gastrointestinal, or hepatobiliary system with systemic metastasis was the most frequently encountered type of cancer. The findings are in line with what has already been established in previous studies of deep vein thrombosis [23]–[25]. Those characteristics of cancer might be useful as a guide to screen occult cancer in patients with cryptogenic stroke.

### Strengths and Weaknesses

We included CA-control patients who had similar extent and histology of cancer to CA-stroke patients. Therefore, the possible effects of cancer itself in elevating D-dimer levels can be eliminated. In addition, we excluded patients who had possible conditions that could raise levels of D-dimer. That may allow us to assume that elevated D-dimer concentrations are a CA-stroke-specific finding.

It is important to note that this study also had some limitations. First, the results should be interpreted with caution, because they were based on data obtained from a single center. Accordingly, our results need independent confirmation through future studies. Second, not all the patients of the CR-stroke group underwent cancer workup. Screening of occult cancer in patients with CR-stroke was determined by the discretion of attending physicians who have many experiences with CA-stroke and was largely based on the DWI pattern and D-dimer levels. Therefore, it may be difficult to generalize our results to all settings even though the ROC curve was good and we found occult cancer in all patients in the CR-stroke group who had ischemic lesions in multiple vascular territories and high D-dimer levels. More systemic approaches are needed to avoid missing hidden malignancies. Third, although all the included subjects underwent cardiologic evaluation including standard electrocardiography, Holter and/or telemetry monitoring and transthoracic echocardiography, transesophageal echocardiography was not performed in all patients, mainly due to coagulopathy, bleeding tendency, mental change, or acute illness related to systemic cancer or stroke, itself. Therefore, it may be possible that some cardioembolic sources with high primary risk for ischemic stroke could not be detected in this study.

### Conclusion

In conclusion, acute ischemic lesions involving multiple vascular territories on DWI and plasma D-dimer levels are independent predictors for CA-stroke. Those laboratory and radiologic features also serve as clues to occult cancer in patients with cryptogenic stroke but without active cancer at the time of stroke. Future study is warranted to optimize strategies for diagnosis and treatment of occult cancer in those patients.
